# Associations between pain, anxiety and depression and mindfulness in patients with burning mouth syndrome: a cross-sectional study

**DOI:** 10.22514/jofph.2025.053

**Published:** 2025-09-12

**Authors:** Jing He, Junjiang Liu, Xin Ye, Mingjia Hu, Ning Xiao, Jia Li, Yansong Song, Fanglong Wu, Fan Liu

**Affiliations:** ^1^State Key Laboratory of Oral Diseases & National Center for Stomatology & National Clinical Research Center for Oral Diseases & Frontier Innovation Center for Dental Medicine Plus, Department of Oral Medicine, West China Hospital of Stomatology, Sichuan University, 610041 Chengdu, Sichuan, China; ^2^State Key Laboratory of Oral Diseases & National Center for Stomatology & National Clinical Research Center for Oral Diseases, Department of Emergency, West China Hospital of Stomatology, Sichuan University, 610041 Chengdu, Sichuan, China; ^3^State Key Laboratory of Oral Diseases & National Center for Stomatology & National Clinical Research Center for Oral Diseases, Department of Nursing, West China Hospital of Stomatology, Sichuan University, 610041 Chengdu, Sichuan, China; ^4^Nursing Key Laboratory of Sichuan Province, Sichuan University, 610041 Chengdu, Sichuan, China

**Keywords:** Burning mouth syndrome, Mindfulness, Pain, Anxiety, Depression

## Abstract

**Background**: Burning mouth syndrome (BMS) is a chronic pain disorder affecting the oral mucosa, often accompanied by psychological comorbidities. Higher levels of mindfulness have been associated with reduced pain and fewer emotional symptoms in some chronic pain conditions, but its role in BMS remains inadequately explored. **Methods**: 146 patients diagnosed with BMS, according to the International Classification of Orofacial Pain, 1st edition, were recruited from the Department of Oral Medicine at a stomatology hospital. Mindfulness, pain intensity and psychological symptoms were assessed using the Five Facet Mindfulness Questionnaire, visual analog scale, and self-report screening tools, respectively. Spearman’s correlation and multiple regression analyses were conducted to evaluate the relationships between mindfulness and levels of pain, anxiety, and depression. **Results**: Mindfulness showed significant negative correlations with pain (*r* = −0.204, *p* < 0.05), anxiety (*r* = −0.309, *p* < 0.01), and depression (*r* = −0.299, *p* < 0.01). After controlling for confounding variables, higher overall mindfulness remained significantly associated with lower pain intensity (β = −0.268, *p* < 0.05), although the associations with anxiety and depression were no longer statistically significant (*p* > 0.05). Among the mindfulness facets, higher scores on the “describing” facet were associated with reduced pain intensity (β = −0.231, *p* < 0.05). Additionally, higher scores on the “non-judging” (Odds Ratio (OR) = 0.871, *p* < 0.05) and “non-reactivity” (OR = 0.869, *p* < 0.05) facets were associated with fewer anxiety symptoms, while the “acting-with-awareness” facet was significantly associated with fewer depressive symptoms (OR = 0.869, *p* < 0.05). **Conclusions**: The overall mindfulness level and the “describing” facet appear to be associated with pain severity, whereas the “non-judging” and “non-reactivity” facets are related to anxiety symptoms, and the “acting-with-awareness” facet is linked to depressive symptoms, suggesting that mindfulness-based interventions may offer a beneficial approach in the management of BMS.

## 1. Introduction

Burning mouth syndrome (BMS) is a chronic pain condition characterized by an unpleasant burning sensation in the oral mucosa [1]. According to the first edition of the International Classification of Orofacial Pain (ICOP) [2], BMS is defined as “an intraoral burning or dysesthetic sensation, recurring daily for more than two hours per day for more than three months, without evident causative lesions on clinical examination and investigation”. The global prevalence of BMS is estimated at 1.73% in the general population and 7.72% in dental clinical settings [3], with a significant predominance among menopausal and postmenopausal women [4]. Despite increasing awareness, the etiology of BMS remains poorly understood, presenting challenges in clinical diagnosis and effective management [5]. Moreover, BMS has been associated with various neurological and psychological factors [6, 7], among which anxiety and depression are the most commonly reported psychological disturbances [8, 9]. The combination of persistent pain and emotional distress significantly impairs patients’ quality of life and psychological well-being [10].

Psychologically oriented interventions have demonstrated clinical benefits in individuals with chronic pain, with mindfulness emerging as one of the most widely employed approaches [11]. Mindfulness-based interventions (MBIs) have been shown to be effective in alleviating symptoms in several chronic pain conditions [12, 13], with a similar benefit in patients with chronic neuropathic pain [11]. Notably, even brief MBIs have been reported to exert meaningful reductions in both pain intensity and negative affect, such as in patients with chronic low back pain [14]. Meanwhile, MBIs have also been proposed for use in the management of oral diseases and chronic orofacial pain [15, 16], offering several advantages, including safety, ease of application, and the absence of drug-related adverse effects. Their practical and non-invasive nature renders them suitable for both patients and clinicians [17]. Conceptually, mindfulness involves cultivated awareness and a nonjudgmental acceptance of internal experiences [18].

The Five Facet Mindfulness Questionnaire (FFMQ) is among the most widely used instruments for assessing mindfulness in clinical and research settings [19]. Previous studies utilizing the FFMQ have reported significant negative correlations between mindfulness levels and both pain severity and emotional symptoms, such as anxiety and depression [20, 21]. However, recent evidence from patients with chronic orofacial pain, including those with trigeminal neuralgia, persistent idiopathic facial pain, or multiple diagnoses, suggests that while mindfulness is inversely correlated with anxiety and depression, it may not be significantly associated with pain intensity or disability [22]. In these studies, the non-judging facet of mindfulness has emerged as the only subscale independently associated with both pain-related disability and the severity of emotional symptoms [23]. Additionally, in patients with temporomandibular disorders, differential associations were observed between specific mindfulness facets and the perception of pain [24].

Despite growing evidence on the relationship between mindfulness and chronic pain, the specific associations among mindfulness, pain intensity, anxiety, and depression in patients with BMS remain poorly defined. Therefore, the present study aimed to assess the level of mindfulness using the FFMQ and its associations with pain, anxiety and depression in individuals with BMS for the potential clinical application of MBIs in this population.

## 2. Materials and methods

### 2.1 Study design and participants

This cross-sectional study was approved by the Ethics Committee of the West China Hospital of Stomatology, Sichuan University (Approval No. WCHSIRB-D-2021-481). Written informed consent was obtained from all participants before study enrollment.

Participants were recruited using a convenience sampling method. Patients diagnosed with BMS at the Department of Oral Medicine, West China Hospital of Stomatology, Sichuan University, between May 2022 and March 2023 were included. The diagnosis of BMS was based on the criteria outlined in the first edition of the ICOP [2], which include the following: (1) oral pain meeting criteria (2) and (3); (2) pain recurring daily for more than two hours per day for a duration exceeding three months; (3) pain characterized by a burning quality and superficial sensation localized to the oral mucosa; (4) oral mucosa with a normal clinical appearance, with exclusion of local or systemic causes; and (5) symptoms not better accounted for by another ICOP or the International Classification of Headache Disorders, 3rd edition (ICHD-3) diagnosis.

The inclusion criteria were as follows: (1) met the diagnostic criteria for BMS; (2) voluntary participation; (3) ability to communicate effectively and comprehend the questionnaire content; and (4) aged ≥18 years.

The exclusion criteria were: (1) concurrent oral mucosal diseases; (2) presence of local irritative factors in the oral cavity, including but not limited to poor restorations, retained roots, residual crowns, moderate to severe periodontitis, or fungal infections; (3) systemic conditions such as anemia, diabetes mellitus, rheumatoid arthritis, or those requiring long-term medication for other illnesses; (4) diseases affecting adjacent organs; (5) a history of diagnosed mental or psychological disorders and the use of anti-anxiety or antidepressant medications, or receipt of psychological therapy within the month preceding study participation; and (6) prior experience with mindfulness meditation.

### 2.2 Data collection

After the participants completed their clinical consultations, a dedicated researcher distributed a paper-based version of the questionnaire and guided them in completing it. Before administering the questionnaire, the researcher offered standardized instructions and obtained consent after explaining the completion requirements, principles of confidentiality, the purpose of the study, and relevant precautions. Participants completed the questionnaire according to their actual condition. If any uncertainties arose during completion, the researcher offered consistent explanations using standardized language. All questionnaires were collected on-site, carefully reviewed and systematically organized by the researcher. Questionnaires with missing items that could not be resolved were deemed invalid.

#### 2.2.1 Demographic and psychosocial information

Demographic and psychosocial information was collected by the research team, including sex (male or female), age, education level (categorized as junior high school or less, high school to college, and bachelor’s degree or higher), marital status (unmarried, married or divorced/widowed), and occupation category (desk work, physically demanding work, unemployed or retired). Information on the duration of BMS symptoms and history of stressful life events in the preceding year was also obtained. A stressful life event was defined as an event that can cause psychological stress in individuals [25].

#### 2.2.2 Pain assessment

A 10-cm visual analogue scale (VAS) was used to evaluate the mean daily pain experienced by participants at the time of their clinical visit. The procedure involved drawing a 10 cm horizontal line on paper, where 0 represented “no burning sensation” and 10 represented an “unbearable burning sensation”. Participants were asked to mark the point on the line that best reflected their perceived pain intensity. The distance from the zero point to the participant’s mark was measured to determine the pain score [26]. VAS is widely recognized as a simple and effective tool for quantifying pain severity and is one of the primary instruments used to evaluate pain in patients with BMS [27].

#### 2.2.3 Anxiety and depression assessment

The level of anxiety symptoms was assessed using the Generalized Anxiety Disorder-7 (GAD-7) scale [28], and the level of depression symptoms was evaluated using the Patient Health Questionnaire-9 (PHQ-9) [29]. These concise and validated self-report tools can be used to evaluate symptoms experienced over the past two weeks, whereby each item is scored on a scale from 0 to 3 points (0 = not at all, 1 = several days, 2 = more than half the days, 3 = nearly every day), with the total score being the sum of all item scores. Higher scores indicate more severe symptoms of anxiety or depression [30].

The GAD-7 consists of seven items, with total scores ranging from 0 to 21, and threshold scores of 5, 10 and 15 corresponding to mild, moderate and severe anxiety symptoms, respectively [31]. The PHQ-9 consists of nine items, with total scores ranging from 0 to 27, and cutoff scores of 5, 10, 15 and 20 indicate mild, moderate, moderately severe, and severe depressive symptoms, respectively [31]. These two scales were used independently to assess anxiety and depression in patients with BMS [32].

For binary logistic regression analysis, a cut-off score of 5 on both the PHQ-9 and GAD-7 was used. Since mild symptoms may not meet the criteria for moderate or severe conditions, they could serve as early indicators of emerging mental health concerns, and neglecting such cases may result in missed opportunities for timely intervention [33, 34].

#### 2.2.4 Mindfulness assessment

The FFMQ developed by Baer and Toney was used to assess the participants’ levels of mindfulness [35] and comprises five dimensions: observing (the tendency to attend to both external and internal stimuli), describing (the ability to label internal experiences with words), acting-with-awareness (engaging in conscious, intentional actions rather than behaving automatically or habitually), non-judging (maintaining awareness of thoughts and sensations without evaluation), and non-reactivity (allowing internal experiences to arise and pass without reacting to them) [19]. The questionnaire contains 39 items, each rated on a 5-point Likert scale ranging from 1 (rarely or never true) to 5 (almost always or always true), yielding a total score ranging from 39 to 195. Higher total scores reflect greater levels of mindfulness [36]. The Cronbach’s alpha coefficients were calculated to assess the internal consistency and reliability of each of the five FFMQ subscales in the BMS population.

### 2.3 Statistical analysis

All statistical analyses were conducted using SPSS version 24.0 (IBM Inc., Chicago, IL, USA), with a significance level set at *p* < 0.05. Descriptive statistics were used to summarize the questionnaire scores and demographic and psychosocial characteristics. Continuous variables were presented as mean ± standard deviation, while categorical variables were reported as frequencies and percentages.

Spearman’s correlation analysis was employed to examine the relationships between mindfulness and levels of pain, anxiety, and depression. The Spearman’s rank correlation coefficient (*r*) ranges from −1 to 1, where 0 indicates no correlation, +1 indicates a perfect positive correlation, and −1 indicates a perfect negative correlation.

Pain, anxiety, and depression levels were each used as dependent variables. Demographic and psychosocial characteristics were included as covariates to assess the associations between the total mindfulness score or each of the five mindfulness facets and the three dependent variables. Prior to regression analyses, normality and homogeneity of variance were assessed. If the data met assumptions of normality and homogeneity, multiple linear regression was performed; otherwise, binary logistic regression analysis was used.

## 3. Results

### 3.1 Demographic and psychosocial information

A total of 148 questionnaires were distributed, of which 146 were deemed valid, resulting in an effective response rate of 98.65%. The demographic and psychosocial characteristics of the patients with BMS are summarized in Table 1.

**Table 1.** Demographic and psychosocial information of patients with burning mouth Syndrome (n = 146).

Variables	Frequency (%)
Age, yr (Mean, SD)	54.53 (9.79)
Sex: Female	131 (89.73)
Education level: Junior high school or less	81 (55.48)
Education level: High school to college	47 (32.19)
Education level: Bachelor’s degree or higher	18 (12.33)
Marital status: Married	133 (91.10)
Marital status: Unmarried	7 (4.79)
Marital status: Divorced or widowed	6 (4.11)
Occupation category: Unemployed or retired	88 (60.27)
Occupation category: Desk work	33 (22.60)
Occupation category: Physically demanding work	25 (17.12)
Stressful life events in the last year: Yes	68 (46.58)
Duration of symptoms of BMS: <1 yr	92 (63.01)

*SD: Standard Deviation; BMS: Burning mouth syndrome.*

### 3.2 Results of screening instruments

In this study, 93 patients (63.70%) exhibited symptoms of anxiety. Of these, 51 (34.93%) had mild anxiety, 27 (18.49%) had moderate anxiety, and 15 (10.27%) had severe anxiety. Additionally, 73 patients (50.00%) presented with symptoms of depression, including 35 (23.97%) with mild depression, 24 (16.44%) with moderate depression, 9 (6.16%) with moderately severe depression and 5 (3.42%) with severe depression.

The internal consistency of each facet of the FFMQ was evaluated using Cronbach’s alpha, which yielded the following results: observing (0.730), describing (0.795), acting-with-awareness (0.837), non-judging (0.705) and non-reactivity (0.645). Overall, these values indicate that the FFMQ facets demonstrated acceptable to good reliability for use in the BMS population. The descriptive statistics for mindfulness, pain intensity, anxiety, and depression are presented in Table 2.

**Table 2.** Descriptive statistics for mindfulness, pain, anxiety and depression in the study population.

Variables	Mean ± SD	Score Range
FFMQ	120.42 ± 12.53	39–195
Observing	18.09 ± 5.89	8–40
Describing	26.07 ± 6.58	8–40
Acting-with-awareness	30.97 ± 6.62	8–40
Non-judging	26.32 ± 5.79	8–40
Non-reacting	18.90 ± 4.89	7–35
VAS	4.86 ± 2.02	0–10
GAD-7 (Med) IQR	6.00 (3.00, 11.00)	0–21
PHQ-9 (Med) IQR	4.50 (2.00, 10.00)	0–27

*SD: Standard Deviation; IQR: Interquartile Range; FFMQ: Five Facet Mindfulness Questionnaire; VAS: visual analogue scale; GAD-7: Generalized Anxiety Disorder-7; PHQ-9: Patient Health Questionnaire-9; Med: Median.*

### 3.3 Correlation analysis between pain, anxiety, depression, and mindfulness

The Spearman correlation analysis examining the relationships among pain, anxiety, depression, mindfulness and its five facets in patients with BMS are presented in Table 3. Nonparametric Spearman correlations were used because the data did not fully meet the assumptions required for Pearson correlation analysis. A significant negative correlation was observed between overall mindfulness and pain intensity (*r* = −0.204, *p* < 0.05), anxiety (*r* = −0.309, *p* < 0.01), and depression (*r* = −0.299, *p* < 0.01). Regarding the five mindfulness facets, the observing facet was positively correlated with both anxiety (*r* = 0.249, *p* < 0.01) and depression (*r* = 0.225, *p* < 0.01), while non-judging facet showed negative correlations with anxiety (*r* = −0.271, *p* < 0.01) and depression (*r* = −0.173, *p* < 0.05). The describing facet was negatively correlated with pain (*r* = −0.177, *p* < 0.05). The acting-with-awareness facet was negatively correlated with pain (*r* = −0.173, *p* < 0.05), anxiety (*r* = −0.509, *p* < 0.01) and depression (*r* = −0.482, *p* < 0.01).

**Table 3.** Spearman correlations among pain, anxiety, depression and mindfulness in patients with BMS.

Variables	VAS	GAD-7	PHQ-9	FFMQ	Observing	Describing	Acting-with-awareness	Non-judging	Non-reacting
VAS	1.000	0.209*	0.238**	−0.204*	0.037	−0.177*	−0.173*	−0.002	−0.052
GAD-7		1.000	0.671**	−0.309**	0.249**	−0.068	−0.509**	−0.271**	−0.098
PHQ-9			1.000	−0.299**	0.225**	−0.112	−0.482**	−0.173*	−0.073
FFMQ				1.000	0.271**	0.751**	0.533**	0.084	0.343**
Observing					1.000	0.249**	−0.467**	−0.496**	0.397**
Describing						1.000	0.260**	−0.213**	0.176*
Acting-with-awareness							1.000	0.313**	−0.117
Non-judging								1.000	−0.400**
Non-reacting									1.000

*: p < 0.05; **: p < 0.01; VAS: visual analogue scale; GAD-7: Generalized Anxiety Disorder-7; PHQ-9: Patient Health Questionnaire-9; FFMQ: Five Facet Mindfulness Questionnaire.

### 3.4 Regression analyses of mindfulness and the level of symptoms of pain, anxiety and depression

To further examine the relationship between mindfulness and the levels of symptoms of pain, anxiety, and depression in patients with BMS, regression analyses were conducted while controlling for relevant covariates, such as sex, age, education level, marital status, employment status, duration of illness, presence of stressful life events in the past year, and overall mindfulness, with pain, anxiety and depression included as dependent variables. The analysis revealed that higher levels of overall mindfulness were significantly associated with lower pain intensity (β = −0.268, *p* < 0.05). However, the associations between mindfulness and the level of symptoms of anxiety and depression were not statistically significant (*p* > 0.05). The detailed results of this analysis are presented in **Supplementary Table 1**.

In addition, a separate regression analysis was conducted to explore the relationships between the five specific facets of mindfulness (observing, describing, acting-with-awareness, non-judging and non-reactivity) and pain intensity, anxiety symptoms, and depressive symptoms, which was also adjusted for the aforementioned covariates (**Supplementary Table 2**). The results showed that a higher score on the describing facet was significantly associated with lower pain intensity (β = −0.231, *p* < 0.05). For anxiety symptoms, higher levels of non-judging (OR = 0.871, 95% Confidence Interval (CI): 0.777–0.977; *p* < 0.05) and non-reactivity (OR = 0.869, 95% CI: 0.770–0.981; *p* < 0.05) were both associated with approximately a 13% reduction in the odds of experiencing mild to severe anxiety symptoms compared to having no or minimal symptoms. Regarding depression, a higher level of acting-with-awareness (OR = 0.869, 95% CI: 0.786–0.960; *p* < 0.05) was similarly associated with about a 13% reduction in the odds of exhibiting mild to severe depressive symptoms compared to no or minimal signs of depression.

## 4. Discussion

To our knowledge, this is the first study to report mindfulness levels in patients with BMS and to examine their associations with pain, anxiety, and depression. Our findings indicate that higher mindfulness levels are correlated with lower pain intensity. After adjusting for potential confounding variables, mindfulness emerged as an independent negative predictor of pain intensity. Previous studies have shown that the relationship between mindfulness and pain intensity varies across populations. For instance, Poulin *et al*. [37] reported that mindfulness negatively predicted pain intensity in cancer survivors with chronic neuropathic pain. Similarly, Nigol *et al*. [36] found that higher mindfulness levels were associated with lower pain severity in individuals with chronic pain, while Greenberg *et al*. [22] observed no significant correlation between mindfulness and pain intensity. We believe that these discrepancies could be attributed to differences in pain processing mechanisms across various disease conditions [38]. Moreover, our study demonstrated that the describing facet of mindfulness was independently associated with reduced pain intensity, suggesting that patients who are better able to articulate their internal experiences may be more capable of redirecting attention away from their pain. This finding aligns with a study by Elvery *et al*. [39], which showed that the describing facet was a negative predictor of pain intensity in university students with chronic or intermittent pain.

In our study, after controlling for confounding variables, the associations between overall mindfulness and the levels of symptoms of anxiety and depression were not statistically significant. This finding differs from previous studies [22, 40], and such inconsistencies may be due to variations in sample characteristics or the specific covariates controlled for in different analyses. Nevertheless, Pleman *et al*. [41] suggested that mindfulness may moderate the impact of fibromyalgia on anxiety symptoms, though its moderating effect on depression appeared less substantial. Further analysis of individual mindfulness facets revealed that higher scores in non-judging and non-reactivity were associated with lower levels of anxiety symptoms, while higher acting-with-awareness was associated with lower levels of depressive symptoms. A recent meta-analysis found that the acting-with-awareness and non-judging facets had strong negative correlations with affective symptoms, while the describing and non-reactivity facets showed moderate associations [42]. Additionally, non-judging was the only facet found to be independently associated with pain-related disability, anxiety, and depression in individuals with chronic orofacial pain [23]. These findings suggest that the relationship between mindfulness facets and psychological symptoms may differ depending on disease type and patient population [42]. In summary, the impact of mindfulness and its facets on pain and anxiety/depression varies depending on the population and disease type.

In our study, patients with higher scores on the observing facet exhibited higher levels of symptoms of anxiety and depression, although these associations were not significant after adjusting for confounding variables. Similar findings have been reported in previous research, where the observing facet was linked to increased pain-related disability and anxiety in patients with chronic orofacial pain [23]. Additionally, a study by Baer *et al*. [35] found a positive correlation between the observing facet and emotional symptoms in individuals without prior mindfulness training. Without the complementary mindfulness skills cultivated through formal training, such as non-judging and non-reactivity, an increased tendency to attend to internal and external stimuli may not result in reduced psychological distress [42]. In the present study, none of the BMS patients had experience in mindfulness practice, suggesting that their observational tendencies were untrained. This untrained attention may lead to excessive focus on oral sensations, potentially exacerbating both pain perception and emotional distress. For example, repeated tongue inspection during self-examination may provoke fear of serious illness, such as cancer, thereby intensifying the psychological burden [43]. Conversely, some evidence suggests that when appropriately trained, observational awareness may enhance emotional regulation and reduce psychological symptoms [44]. The Psychological Flexibility Model, which comprises six core processes (including acceptance, cognitive defusion, present-moment awareness, self-as-context, values, and committed action), has been widely applied in chronic pain management as it may offer a useful framework for improving observational skills in BMS patients as part of MBIs [45].

We also found that the average age of BMS patients was 54.53 years, with a predominance of females (89.73%), indicating that middle-aged and elderly women represent the primary population affected by BMS [46, 47]. Stressful life events are known to trigger or exacerbate both pain and psychological disturbances in patients with BMS [48]. In our study, nearly half of the patients (46.58%) reported experiencing stressful life events within the past year, highlighting the need to address emotional well-being in addition to somatic symptoms when managing BMS. Due to the persistent nature of chronic pain and the presence of negative emotional states such as anxiety and depression, BMS can significantly impair both physical and psychological health, ultimately reducing patients’ quality of life [49]. However, patient management remains challenging [50, 51], and in this regard, psychological interventions, including cognitive-behavioral therapy and group psychotherapy, have been shown to alleviate both physical and psychological distress in this population [5]. MBIs, as a form of psychological therapy, have also demonstrated benefits in managing chronic pain disorders [11, 12, 13, 14]. Moreover, our findings showed that in BMS patients, overall mindfulness and the describing facet were associated with lower pain intensity, while the non-judging and non-reactivity facets were linked to lower levels of anxiety symptoms, and the acting-with-awareness facet was associated with lower levels of depressive symptoms. These results suggest that MBIs may hold promise as a complementary approach for managing BMS, offering new possibilities for improving patient outcomes.

However, several limitations of this study should be acknowledged. First, the cross-sectional design precludes any inference of causality regarding the effects of mindfulness on pain, emotional symptoms or quality of life. Second, the study focused solely on pain symptoms and did not explore the potential associations between mindfulness and other abnormal oral sensations commonly reported in BMS, such as numbness, itching, or xerostomia. Third, the relationships among pain, anxiety, depression and mindfulness may have been influenced by unmeasured variables such as pain catastrophizing and pain interference, which were not included in our analyses. Future studies are warranted to investigate these additional psychological factors and to confirm the robustness of the observed associations. Finally, due to the limited sample size, we controlled for sociodemographic variables in the regression models to account for potential confounding, although this may have reduced the statistical power of the analyses. Future research should consider larger sample sizes or adopt causal inference methodologies to further validate and clarify the relationships among mindfulness, pain, and emotional symptoms in BMS.

## 5. Conclusions

Higher levels of mindfulness were associated with lower pain intensity in BMS patients. Specifically, overall mindfulness and the describing facet were negatively associated with pain, while the non-judging and non-reactivity facets were related to lower levels of anxiety symptoms, and the acting-with-awareness facet was linked to reduced depressive symptoms. These findings suggest that MBIs may offer a promising approach to the management of BMS. Future research should focus on identifying appropriate formats of MBIs tailored to the BMS population, evaluating their clinical effectiveness, and investigating additional influencing factors to further clarify the role of mindfulness in the comprehensive management of BMS.

## Supplementary Material

Supplementary material associated with this article can be found, in the online version, at https://files.jofph.com/ files/article/1966372479604539392/attachment/ Supplementary%20material.docx.


