# Supplementary material for: Associations between pain, anxiety and depression and mindfulness in patients with burning mouth syndrome: a cross-sectional study
Source: J Oral Facial Pain Headache. 2025 Sep 12;39(3):113–20. doi: 10.22514/jofph.2025.053 (PMC12520433; doi:10.22514/jofph.2025.053)
Supplement: Supplementary file 1 [file Supplementary-material.docx]

Supplementary material

Supplementary Table 1. Regression analyses of mindfulness and the level of symptoms of pain, anxiety and depression in patients with BMS.

| Predictor variables | | Outcome variables | | | | | | | | | | | | | | |
| --- | --- | --- | --- | --- | --- | --- | --- | --- | --- | --- | --- | --- | --- | --- | --- | --- |
|  |  | VAS^d,†^ | | | | | GAD-7: Mild-to-Severe Anxiety^b,‡^ | | | | | PHQ-9: Mild-to-Severe Depression^c,‡^ | | | | |
|  |  | B | β | 95% CI | | *p* | B | OR | 95% CI | | *p* | B | OR | 95% CI | | *p* |
| Sex | | | | | | | | | | | | | | | | |
|  | Male | Ref |  |  |  |  | Ref |  |  |  |  | Ref |  |  |  |  |
|  | Female | 0.375 | 0.056 | −0.744 | 1.494 | 0.509 | −0.219 | 0.803 | 0.197 | 3.275 | 0.760 | 0.467 | 1.595 | 0.398 | 6.400 | 0.510 |
| Marital status | | | | | | | | | | | | | | | | |
|  | Unmarried | Ref |  |  |  |  | Ref |  |  |  |  | Ref |  |  |  |  |
|  | Married | −0.124 | −0.018 | −1.838 | 1.589 | 0.886 | 1.460 | 4.307 | 0.555 | 33.428 | 0.163 | −2.990 | 0.050 | 0.004 | 0.602 | 0.018* |
|  | Divorced or widowed | 0.102 | 0.010 | −2.260 | 2.464 | 0.932 | −0.180 | 0.835 | 0.039 | 18.053 | 0.909 | −1.978 | 0.138 | 0.005 | 4.032 | 0.250 |
| Occupation category | | | | | | | | | | | | | | | | |
|  | Desk work | Ref |  |  |  |  | Ref |  |  |  |  | Ref |  |  |  |  |
|  | Physically demanding work | −0.573 | −0.107 | −1.734 | 0.589 | 0.331 | −0.641 | 0.527 | 0.106 | 2.627 | 0.434 | 0.306 | 1.357 | 0.298 | 6.191 | 0.693 |
|  | Unemployed or retired | 0.184 | 0.045 | −0.796 | 1.164 | 0.710 | −0.522 | 0.593 | 0.156 | 2.261 | 0.444 | −0.755 | 0.470 | 0.133 | 1.663 | 0.242 |
| Duration of symptoms | | | | | | | | | | | | | | | | |
|  | <1 yr | Ref |  |  |  |  | Ref |  |  |  |  | Ref |  |  |  |  |
|  | ≥1 yr | 0.277 | 0.066 | −0.411 | 0.965 | 0.428 | 0.109 | 1.115 | 0.435 | 2.858 | 0.821 | 0.171 | 1.187 | 0.499 | 2.821 | 0.698 |
| Stressful life events | | | | | | | | | | | | | | | | |
|  | No | Ref |  |  |  |  | Ref |  |  |  |  | Ref |  |  |  |  |
|  | Yes | −0.127 | −0.031 | −0.846 | 0.592 | 0.727 | −1.758 | 0.172 | 0.064 | 0.464 | 0.001** | 0.389 | 1.475 | 0.599 | 3.633 | 0.398 |
| Age | | 0.007 | 0.032 | −0.032 | 0.045 | 0.735 | −0.001 | 0.999 | 0.949 | 1.052 | 0.972 | 0.054 | 1.055 | 1.000 | 1.113 | 0.048* |
| Education level^#^ | | 0.551 | 0.197 | −0.092 | 1.194 | 0.092 | 0.096 | 1.100 | 0.484 | 2.499 | 0.819 | −0.482 | 0.617 | 0.268 | 1.420 | 0.257 |
| FFMQ^a^ | | −0.043 | −0.268 | −0.073 | −0.013 | 0.005** | −0.038 | 0.963 | 0.922 | 1.004 | 0.078 | −0.025 | 0.976 | 0.938 | 1.015 | 0.226 |
| GAD-7^b^ | | 0.008 | 0.002 | −0.846 | 0.863 | 0.984 |  |  |  |  |  | 2.788 | 16.250 | 5.647 | 46.764 | 0.001** |
| PHQ-9^c^ | | 0.483 | 0.120 | −0.331 | 1.297 | 0.243 | 2.832 | 16.979 | 5.884 | 48.994 | 0.001** |  |  |  |  |  |
| VAS^d^ | |  |  |  |  |  | −0.011 | 0.989 | 0.778 | 1.257 | 0.927 | 0.110 | 1.117 | 0.900 | 1.385 | 0.316 |

Note: **p* < 0.05, ***p* < 0.01.

B: Unstandardized coefficients; β: Standardized coefficient; CI: Confidence interval; OR: Odds ratio; Ref: Reference; VAS: visual analogue scale; GAD-7: Generalized Anxiety Disorder-7; PHQ-9: Patient Health Questionnaire-9; FFMQ: Five Facet Mindfulness Questionnaire; yr: year.

^a^: Five Facet Mindfulness Questionnaire; ^b^: Generalized Anxiety Disorder-7; ^c^: Patient Health Questionnaire-9; ^d^: Visual Analogue Scale.

^†^: multivariate linear regression analysis (VAS residual conforms to normal distributions and homogeneity of variance).

^‡^: binary logistic analyses (GAD-7 and PHQ-9 residuals do not conform to normal distributions).

^#^: “Education level” has been included in regression models as a continuous (ordinal) variable.

Supplementary Table 2. Regression analyses of the five facets of mindfulness and the levels of pain, anxiety and depression in patients with BMS.

| Predictor variables | | Outcome variables | | | | | | | | | | | | | | |
| --- | --- | --- | --- | --- | --- | --- | --- | --- | --- | --- | --- | --- | --- | --- | --- | --- |
|  |  | VAS^c,†^ | | | | | GAD-7: Mild-to-Severe Anxiety^a,‡^ | | | | | PHQ-9: Mild-to-Severe Depression^b,‡^ | | | | |
|  |  | Β | β | 95% CI | | *p* | B | OR | 95% CI | | *p* | B | OR | 95% CI | | *p* |
| Sex | | | | | | | | | | | | | | | | |
|  | Male | Ref |  |  |  |  | Ref |  |  |  |  | Ref |  |  |  |  |
|  | Female | 0.578 | 0.087 | −0.573 | 1.729 | 0.322 | −0.183 | 0.833 | 0.186 | 3.735 | 0.811 | 0.781 | 2.183 | 0.486 | 9.814 | 0.309 |
| Marital status | | | | | | | | | | | | | | | | |
|  | Unmarried | Ref |  |  |  |  | Ref |  |  |  |  | Ref |  |  |  |  |
|  | Married | −0.189 | −0.027 | −1.937 | 1.560 | 0.831 | 2.023 | 7.560 | 0.877 | 65.187 | 0.066 | −3.344 | 0.035 | 0.003 | 0.397 | 0.007** |
|  | Divorced or widowed | 0.352 | 0.035 | −2.047 | 2.750 | 0.772 | −0.353 | 0.702 | 0.026 | 19.052 | 0.834 | −1.952 | 0.142 | 0.004 | 4.774 | 0.276 |
| Occupation category | | | | | | | | | | | | | | | | |
|  | Desk work | Ref |  |  |  |  | Ref |  |  |  |  | Ref |  |  |  |  |
|  | Physically demanding work | −0.718 | −0.134 | −1.917 | 0.481 | 0.238 | −0.306 | 0.736 | 0.139 | 3.908 | 0.719 | 0.397 | 1.488 | 0.264 | 8.387 | 0.652 |
|  | Unemployed or retired | 0.069 | 0.017 | −0.933 | 1.072 | 0.891 | −0.391 | 0.677 | 0.153 | 2.989 | 0.606 | −0.765 | 0.465 | 0.120 | 1.802 | 0.268 |
| Duration of symptoms | | | | | | | | | | | | | | | | |
|  | <1 yr | Ref |  |  |  |  | Ref |  |  |  |  | Ref |  |  |  |  |
|  | ≥1 yr | 0.225 | 0.054 | −0.475 | 0.926 | 0.525 | 0.075 | 1.078 | 0.383 | 3.035 | 0.887 | 0.173 | 1.189 | 0.473 | 2.988 | 0.713 |
| Stressful life events | | | | | | | | | | | | | | | | |
|  | No | Ref |  |  |  |  | Ref |  |  |  |  | Ref |  |  |  |  |
|  | Yes | −0.151 | −0.037 | −0.883 | 0.581 | 0.683 | −1.740 | 0.176 | 0.062 | 0.499 | 0.001** | 0.620 | 1.859 | 0.703 | 4.915 | 0.212 |
| Age | | 0.009 | 0.043 | −0.031 | 0.048 | 0.655 | −0.013 | 0.988 | 0.936 | 1.042 | 0.648 | 0.053 | 1.054 | 0.998 | 1.114 | 0.061 |
| Education level^#^ | | 0.588 | 0.210 | −0.061 | 1.237 | 0.075 | 0.194 | 1.214 | 0.493 | 2.989 | 0.673 | −0.371 | 0.690 | 0.281 | 1.695 | 0.419 |
| Observing | | 0.014 | 0.039 | −0.064 | 0.091 | 0.729 | 0.025 | 1.025 | 0.916 | 1.147 | 0.664 | 0.000 | 1.000 | 0.904 | 1.107 | 0.996 |
| Describing | | −0.071 | −0.231 | −0.134 | −0.008 | 0.028* | −0.016 | 0.984 | 0.897 | 1.079 | 0.731 | 0.011 | 1.011 | 0.926 | 1.104 | 0.810 |
| Acting-with-awareness | | −0.041 | −0.133 | −0.111 | 0.029 | 0.253 | −0.042 | 0.959 | 0.865 | 1.063 | 0.428 | −0.141 | 0.869 | 0.786 | 0.960 | 0.006** |
| Non-judging | | 0.002 | 0.006 | −0.076 | 0.080 | 0.961 | −0.138 | 0.871 | 0.777 | 0.977 | 0.018* | 0.069 | 1.071 | 0.966 | 1.187 | 0.193 |
| Non-reacting | | −0.052 | −0.124 | −0.135 | 0.032 | 0.223 | −0.140 | 0.869 | 0.770 | 0.981 | 0.023* | 0.014 | 1.014 | 0.909 | 1.131 | 0.804 |
| GAD-7^a^ | | 0.025 | 0.006 | −0.856 | 0.906 | 0.956 |  |  |  |  |  | 2.843 | 17.165 | 5.466 | 53.900 | 0.001** |
| PHQ-9^b^ | | 0.386 | 0.096 | −0.460 | 1.232 | 0.369 | 2.904 | 18.255 | 5.673 | 58.742 | 0.001** |  |  |  |  |  |
| VAS^c^ | |  |  |  |  |  | 0.015 | 1.016 | 0.793 | 1.301 | 0.903 | 0.091 | 1.095 | 0.878 | 1.366 | 0.419 |

Note: **p* < 0.05, ***p* < 0.01.

B: Unstandardized coefficients; β: Standardized coefficient; CI: Confidence interval; OR: Odds ratio; Ref: Reference; VAS: visual analogue scale; GAD-7: Generalized Anxiety Disorder-7; PHQ-9: Patient Health Questionnaire-9; yr: year.

^a^: Generalized Anxiety Disorder-7; ^b^: Patient Health Questionnaire-9; ^c^: Visual Analogue Scale.

^†^: multivariate linear regression analysis (VAS residual conforms to normal distributions and homogeneity of variance).

^‡^: binary logistic analyses (GAD-7 and PHQ-9 residuals do not conform to normal distributions).

^#^: “Education level” has been included in regression models as a continuous (ordinal) variable.
